# Exploring the differences in mortality and its associated factors among young-old and old-old COVID-19 patients

**DOI:** 10.3389/fmed.2025.1608667

**Published:** 2025-07-30

**Authors:** Linyi Zhong, Linlin Huang, Mengchen Zhang, Congcong Tian, Lijuan Zhang, Guobin Song

**Affiliations:** ^1^Hebei Medical University, Shijiazhuang, Hebei, China; ^2^Department of General Medicine, Shijiazhuang People's Hospital, Shijiazhuang, Hebei, China

**Keywords:** COVID-19, older adults, mortality, risk factors, retrospective study

## Abstract

**Background:**

This study aims to compare the differences in mortality and related factors between old-old and young-old COVID-19 patients and find unique factors related to survival in old-old patients.

**Study design:**

Single-center retrospective cohort study following STROBE guidelines.

**Methods:**

We included 302 elderly (≥65 years old) COVID-19 patients admitted to Shijiazhuang People’s Hospital from December 1, 2022 to March 31, 2023. Among them, 142 were assigned to the young-old group (65–74 years old) and 160 in the old-old group (≥75 years old). Demographic, clinical and laboratory data were extracted, and descriptive statistical analysis, comparison of differences between groups, Cox proportional hazards regression analysis, and subgroup analysis were adopted.

**Results:**

Compared with the young-old group, the mortality of old-old patients was higher (31.3% vs. 12.7%, *p* < 0.001). Risk factors associated with mortality specifically in old-old patients include dyspnea (HR: 2.829, 95%CI: 1.571–5.093), acute cardiac injury (HR: 2.403, 95%CI: 1.369–4.219), and diabetes (HR: 2.401, 95%CI: 1.311–4.397), glucocorticoid therapy (HR: 2.397, 95%CI: 1.198–4.798). Moreover, there was a significant difference in the survival curves between the young-old and the old-old group (*p* = 0.0001). However, no significant sex differences in mortality and survival curves were found in either group.

**Conclusion:**

This study found for the first time that dyspnea symptoms, acute heart injury, diabetes, and glucocorticoid therapy are unique risk factors related to survival in old-old patients with COVID-19. These factors need more attention when treating old-old patients to prevent poor prognosis.

## 1 Introduction

In January 2020, the World Health Organization declared the novel coronavirus outbreak a Public Health Emergency of International Concern (PHEIC), and subsequently named the disease COVID-19 in February 2020. The global pandemic status was declared in March 2020. As of August 2024, the number of confirmed cases worldwide has reached 776 million, and the death toll is approximately 7.1 million (1). Compared with the general population, the elderly are more susceptible to COVID-19 (2), and the mortality rate among those infected with COVID-19 is higher in the elderly (3). Possible reasons include elderly patients who have low immunity, increased inflammatory response, and aging-related complications such as diabetes and cardiovascular disease (4).

It is worth noting that there is heterogeneity in the elderly population, for example, the prevalence of frailty, the number of comorbidities, and the degree of immune senescence increase with age (5). The age of 75 is used as the classification standard of the older group, and this threshold is also consistent with demographic studies that categorize the “old-old” as individuals aged 75 and above, reflecting a distinct subgroup with higher morbidity and mortality risks (6, 7). In a study on the clinical characteristics of elderly COVID-19 patients by Wei et al., it was found that systemic inflammation, lung and extrapulmonary organ damage were more obvious in old-old COVID-19 patients (>75 years old) (8). More importantly, CJ et al. found that the mortality rate of old-old was higher than that of young-old COVID-19 patients (5). However, these studies just explored age as a factor affecting the overall mortality rate and failed to explore the unique related factors and differences in the mortality of young-old and old-old COVID-19 patients. Exploring the differences in the related factors of mortality risk between young-old and old-old COVID-19 patients will help to better treat elderly patients of different age stratifications and more accurately reduce the mortality rate of elderly COVID-19 patients.

This study aims to explore the differences in mortality rate and related risk factors among young-old COVID-19 patients and old-old COVID-19 patients, and to compare the differences in mortality rate and mortality-related factors between the two groups of elderly patients. Specifically, we aim to divide elderly COVID-19 patients into young-old group and old-old group, then compare the differences in mortality rate and clinical characteristics between the two groups. In addition, we explore the factors related to the risk of death in the two groups and discuss their differences, and further explore the differences in survival curves between the young-old group and the old-old group.

## 2 Research design and methods

### 2.1 Study design

This single-center retrospective study following STROBE guidelines (checklist in Supplementary Material) was conducted in Shijiazhuang People’s Hospital, a government-designated hospital for the treatment of COVID-19. We included 302 elderly (≥65 years old) COVID-19 patients admitted to Shijiazhuang People’s Hospital from December 1, 2022 to March 31, 2023.

Inclusion Criteria:

1) Laboratory-confirmed COVID-19 diagnosis (RT-PCR positive).
2) Age ≥65 years at admission.
3) Hospitalized at Shijiazhuang People’s Hospital between December 1, 2022 and March 31, 2023.
4) Complete medical records including:

- Admission/discharge dates.
- Vital signs at admission.
- Laboratory test results.
- Outcome status (survival/death).

Exclusion Criteria:

1) Concurrent malignant tumors (active chemotherapy/radiotherapy).
2) Hospital discharge within 24 h of admission (typically mild cases).
3) Transfer to other hospitals with indeterminate outcomes.
4) Missing critical data elements (>50% of key variables).

Based on previous studies (9), we divided elderly COVID-19 patients into two age groups, including a young-old COVID-19 group (65–74 years old) and an old-old COVID-19 group (≥75 years old). All elderly patients with novel coronavirus infection included in the study were diagnosed with novel coronavirus infection by their attending physicians on electronic medical records. This study was approved by the Institutional Ethics Committee of Shijiazhuang People’s Hospital (Approval Number: YKLS2024111) in accordance with the Declaration of Helsinki. As a retrospective study using anonymized data, the ethics committee waived the requirement for informed consent. All methods were performed in accordance with relevant guidelines and regulations. A formal sample size calculation was not performed *a priori* given the observational nature of the study and our aim to include available cases during the pandemic phase. However, post-hoc power analysis indicated that with 142 young-old and 160 old-old patients, the study had >80% power (*α* = 0.05, two-tailed) to detect a mortality difference of ≥15% between groups, based on observed event rates (12.7% vs. 31.3%). This effect size is clinically meaningful and aligns with prior studies (10, 11).

### 2.2 Data abstraction

The extracted data included age, sex, Body Mass Index (BMI), other underlying comorbidities (chronic pulmonary disease, chronic kidney disease, hypertension, chronic neurological disease, cerebrovascular disease, cardiovascular disease, diabetes), onset symptoms (cough and expectoration, fever, feel cold, chest pain, chest tightness, myalgia, stomachache, fatigue, palpitate, inappetence, nausea and vomiting, pharyngeal discomfort, dyspnea, stuffy and running nose, confusion), vital signs on admission (temperature, heart rate, respiratory rate, mean arterial pressure), laboratory parameters (blood gas analysis: Power of Hydrogen (PH), Partial pressure of arterial oxygen (PaO_2_), Partial pressure of arterial carbon dioxide (PaCO_2_), Partial pressure of arterial oxygen (PaO₂) to Fraction of inspired oxygen (FiO₂) ratio (PaO_2_/FiO_2_), lactate; blood routine: white blood cell count, platelet count, hemoglobin, lymphocyte count, neutrophil count; coagulation function: D-dimer, Prothrombin Time (PT), Activated Partial Thromboplastin Time (APTT), International Normalized Ratio (INR), Fibrinogen Degradation Products (FDP), Fibrinogen (FIB), Thrombin Time (TT); liver and kidney function: urea, creatinine, Aspartate Transaminase (AST), Alanine Transaminase (ALT), Total Bilirubin (TBIL), albumin; electrolytes: sodium, kalium; cardiac markers: Brain Natriuretic Peptide (BNP), lactate dehydrogenase, creatine kinase, creatine kinase mb, cardiac troponin I; inflammatory indicators: Interleukin-6 (IL6), procalcitonin), complications (acute liver injury, Acute Respiratory Distress Syndrome (ARDS), acute kidney injury, Systemic Inflammatory Response Syndrome (SIRS), acute cardiac injury, respiratory weakness), clinical classification (medium, severe, critical), therapeutic drugs (antiviral drugs, antibiotic drugs, glucocorticoids, anticoagulants, Ig/Tα1 drugs), respiratory support therapy (supplemental oxygen, non-invasive mechanical ventilation, invasive mechanical ventilation), length of hospital stays, and outcomes (survival, death). Data were extracted from the electronic medical record system of Shijiazhuang People’s Hospital. All data were reviewed by experienced doctors.

### 2.3 Definitions

The diagnosis and clinical classification of COVID-19 were carried out in accordance with the 10th edition of the Diagnosis and Treatment Plan for Novel Coronavirus Infection issued by the National Health Commission of China (12). ARDS was defined according to the Berlin definition (13). Acute liver injury was defined as an increase in alanine aminotransferase to more than 3 times the upper reference limit, or an increase in aspartate aminotransferase to more than 3 times the upper reference limit, or an increase in total bilirubin to more than 2 times the upper reference limit, regardless of liver comorbidities (14). SIRS was defined as two or more of the following: abnormal body temperature (>38°C or <36°C), increased respiratory rate (>20 times/min) or PaCO2 < 32 mmHg, increased heart rate (>90 times/min), and irregular white blood cell count (>12 × 10^9/L or <4 × 10^9/L) (15). Acute kidney injury was diagnosed according to the Kidney Disease: Improving Global Outcomes (KDIGO) clinical practice guidelines (16). If the serum level of cardiac biomarkers (such as high-sensitivity cardiac troponin I) is higher than the 99th upper limit of the percentile reference, or the electrocardiogram and echocardiogram show new abnormalities, acute cardiac injury is diagnosed (17). Respiratory weakness is defined as oxygen partial pressure <60 mmHg and/or oxygenation index <300 mmHg and/or blood oxygen saturation <90%. Survival time was calculated from hospital admission until: (a) Death (for deceased patients), or (b) Discharge (for survivors), with censoring at last follow-up (30 days).

The primary outcomes were death outcomes and survival time of elderly COVID-19 patients. Risk factors for mortality were analyzed as explanatory variables rather than outcomes, with their identification serving as the study’s analytical goal rather than an endpoint per se. This approach aligns with our aim to compare age-stratified differences in mortality predictors.

### 2.4 Statistical analysis

We did not make any assumptions about missing data. According to whether the age was ≥75 years old, the subjects were divided into the young-old and old-old groups. Descriptive statistical analysis was performed on the total population and the subgroups. Categorical variables were described as frequencies and percentages, and the chi-square test was used to compare the differences between groups. The Shapiro–Wilk test was used to test the normal distribution of continuous variables. Variables that met the normal distribution were described as means and standard deviations, and the independent sample *t* test was used to compare the differences between groups; variables that were not normally distributed were described as medians and Q1–Q3 intervals, and the Mann–Whitney U test was used to compare the differences between groups.

To explore the risk factors associated with death in young-old and old-old COVID-19 patients, we performed univariate Cox proportional hazard regression models in each group. If the event distribution was not suitable for Cox regression analysis (i.e., chest pain, palpitate, myalgia, stomachache, pharyngeal discomfort, stuffy and running nose, chronic neurological diseases, clinical classification, PH, D-dimer, BNP, Creatine kinase, IL6, antiviral drugs, antibiotic drugs), the variable was excluded from the Cox regression model.

The survival curves of younger and older COVID-19 patients were plotted using the Kaplan Meier method and the log-rank test. In addition, we performed a subgroup analysis based on sex differences to explore the sex differences in mortality in the young-old and old-old groups. Statistical analysis was performed using R (version 4.4.0). A two-sided *p* value of <0.05 was considered statistically significant.

## 3 Results

### 3.1 Characteristics of participants

A total of 302 elderly COVID-19 patients were included, with a median age of 75 years (70.0–81.0), of which 60.3% were male and 39.7% were female. Among them, 142 were in the young-old group (median age was 70 years [68.0–72.0], 64.8% were male), and 160 were in the old-old group (median age was 81.0 years [78.0–86.0], 56.3% were male). In the old-old group, the clinical severity included critical (28.8%), severe (31.9%), and medium (39.3%), while the young-old group had critical (11.3%), severe (34.5%), and medium (54.2%), *p* < 0.001. Within 30 days of follow-up, compared with the young-old group, mortality was higher in the old-old group (31.3% vs. 12.7%, *p* < 0.001), and the incidence of cerebrovascular disease was higher in the old-old group (*p* = 0.010), and were more likely to have symptoms of confusion (*p* = 0.016), and acute cardiac injury (*p* = 0.043). The old-old group exhibited significantly lower levels of the following indicators: platelet count, lymphocyte count, and albumin (all *p* < 0.05). Conversely, these indicators are higher in the old-old group: D-dimer, APTT, FDP, TT, urea, creatinine, AST, BNP, creatine kinase, creatine kinase mb, cardiac troponin I, IL6, procalcitonin (all *p* < 0.05). In terms of treatment, the old-old patients were more likely to receive antibiotic therapy (*p* = 0.004) (see Table 1).

**Table 1:** Characteristics, laboratory findings, complications, comorbidities, treatments, and outcomes of young-old and old-old among senior COVID-19 patients.

Variables	Normal range	Senior COVID-19 patients			
		Total (*n* = 302)	Young-old (*n* = 142)	Old-old (*n* = 160)	*P* value
Sex					0.163
Male, *n* (%)		182 (60.3)	92 (64.8)	90 (56.3)	–
Female, *n* (%)		120 (39.7)	50 (35.2)	70 (43.7)	–
Age, median (IQR)		75.0 (70.0–81.0)	70.0 (68.0–72.0)	81.0 (78.0–86.0)	**<0.001**
Signs and symptoms					
Cough and expectoration, *n* (%)		260 (86.1)	120 (84.5)	140 (87.5)	0.560
Fever, *n* (%)		265 (87.7)	129 (90.8)	136 (85.0)	0.171
Feel cold, *n* (%)		28 (9.3)	13 (9.2)	15 (9.4)	1.000
Chest pain, *n* (%)		5 (1.7)	2 (1.4)	3 (1.9)	1.000
Chest tightness, *n* (%)		39 (12.9)	23 (16.2)	16 (10.0)	0.152
Myalgia, *n* (%)		33 (10.9)	20 (14.1)	13 (8.1)	0.141
Stomachache, *n* (%)		6 (2.0)	4 (2.8)	2 (1.3)	0.575
Fatigue, *n* (%)		98 (32.5)	52 (36.6)	46 (28.8)	0.182
Palpitate, *n* (%)		5 (1.7)	2 (1.4)	3 (1.9)	1.000
Inappetence, *n* (%)		117 (38.7)	57 (40.1)	60 (37.5)	0.725
Nausea and vomiting, *n* (%)		35 (11.6)	16 (11.3)	19 (11.9)	1.000
Pharyngeal discomfort, *n* (%)		31 (10.3)	13 (9.2)	18 (11.3)	0.683
Dyspnea, *n* (%)		152 (50.3)	78 (54.9)	74 (46.3)	0.164
Stuffy and running nose, *n* (%)		13 (4.3)	7 (4.9)	6 (3.8)	0.826
Confusion, *n* (%)		29 (9.6)	7 (4.9)	22 (13.8)	**0.016**
BMI (kg/㎡), median (IQR)		24.2 (21.6–27.1)	25.2 (22.1–27.3)	23.9 (21.3–26.7)	0.078
Temperature (°C), median (IQR)		36.7 (36.5–37.0)	36.7 (36.5–37.2)	36.7 (36.5–37.0)	0.614
Respiratory rate (rpm), median (IQR)		20.0 (19.0–22.0)	20.0 (19.0–22.0)	20.0 (19.0–23.0)	0.168
Mean arterial pressure (mmHg), median (IQR)		98.33 (88.67–109.00)	98.17 (89.84–107.33)	98.33 (87.33–110.33)	0.720
Heart rate (bpm), median (IQR)		82.0 (74.0–96.0)	82.0 (74.0–97.5)	82.0 (74.0–94.0)	0.803
Complications					
Acute liver injury, *n* (%)		35 (11.6)	17 (12.0)	18 (11.3)	0.988
ARDS, *n* (%)		3 (1.0)	1 (0.7)	2 (1.3)	1.000
AKI, *n* (%)		31 (10.3)	10 (7.0)	21 (13.1)	0.122
SIRS, *n* (%)		23 (7.6)	6 (4.2)	17 (10.6)	0.061
Acute cardiac injury, *n* (%)		63 (20.9)	22 (15.5)	41 (25.6)	**0.043**
Respiratory weakness, *n* (%)		139 (46.0)	60 (42.3)	79 (49.4)	0.261
Comorbidities					
Chronic pulmonary disease, *n* (%)		48 (15.9)	26 (18.3)	22 (13.8)	0.355
Chronic kidney disease, *n* (%)		35 (11.6)	12 (8.5)	23 (14.4)	0.154
Hypertension, *n* (%)		176 (58.3)	85 (59.9)	91 (56.9)	0.683
Chronic neurological disease, *n* (%)		28 (9.3)	8 (5.6)	20 (12.5)	0.064
Cerebrovascular disease, *n* (%)		100 (33.1)	36 (25.4)	64 (40.0)	**0.010**
Cardiovascular disease, *n* (%)		130 (43.0)	58 (40.8)	72 (45.0)	0.541
Diabetes, *n* (%)		151 (50.0)	71 (50.0)	80 (50.0)	1.000
Clinical classification					**<0.001**
Medium, *n* (%)		140 (46.4)	77 (54.2)	63 (39.3)	–
Severe, *n* (%)		100 (33.1)	49 (34.5)	51 (31.9)	–
Critical, *n* (%)		62 (20.5)	16 (11.3)	46 (28.8)	–
Laboratory findings					
PH, median (IQR)	7.35–7.45	7.43 (7.40–7.45)	7.42 (7.39–7.45)	7.43 (7.40–7.45)	0.174
PaO2 (mmHg), median (IQR)	75–100	70.5 (61.1–82.5)	71.4 (61.6–82.3)	69.7 (59.4–82.4)	0.550
PaO2/FiO2 (mmHg), median (IQR)	>300	338.26 (279.88–415.00)	336.30 (278.65–406.67)	346.71 (280.25–416.05)	0.667
PaCO2 (mmHg), median (IQR)	32–45	34.4 (31.0–38.0)	34.6 (31.8–38.3)	34.3 (30.6–37.8)	0.474
Lactate (mmol/L), median (IQR)	0.5–2.0	1.76 (1.39–2.17)	1.73 (1.39–2.19)	1.80 (1.39–2.16)	0.735
White blood cell count (×10^9^/L), median (IQR)	3.5–9.5	6.74 (4.97–9.39)	6.79 (5.10–9.15)	6.72 (4.90–10.05)	0.807
Platelet count (×10^9^/L), median (IQR)	125–350	182.0 (140.3–242.5)	200.5 (146.3–268.5)	174.0 (131.8–225.3)	**0.005**
Hemoglobin (g/L), median (IQR)	115–150	126.8 (114.5–138.0)	129.0 (117.0–139.0)	125.0 (111.5–136.3)	0.053
Lymphocyte count (×10^9^/L), median (IQR)	1.1–3.2	0.74 (0.48–1.16)	0.87 (0.60–1.40)	0.66 (0.45–0.98)	**<0.001**
Neutrophil count (×10^9^/L), median (IQR)	1.8–6.3	5.02 (3.57–7.90)	4.90 (3.75–7.15)	5.25 (3.38–8.45)	0.645
D-dimer (ng/mL), median (IQR)	0–1,000	1130.00 (297.44–2319.50)	870.00 (222.18–2000.00)	1400.00 (531.00–2572.50)	**<0.001**
PT (s), median (IQR)	10–14	12.2 (11.2–13.3)	12.0 (11.2–13.0)	12.3 (11.3–13.6)	0.063
APTT (s), median (IQR)	24–39	32.3 (28.7–37.3)	30.8 (28.3–35.6)	33.0 (29.3–38.8)	**0.010**
INR, median (IQR)	0.8–1.2	1.01 (0.95–1.09)	1.02 (0.95–1.08)	1.00 (0.95–1.09)	0.977
FDP (μg/mL), median (IQR)	0–5	4.50 (2.81–7.30)	3.80 (2.69–6.40)	4.90 (3.30–8.05)	**0.022**
FIB (g/L), median (IQR)	2–4	4.21 (3.43–5.12)	4.11 (3.28–5.03)	4.27 (3.58–5.32)	0.294
TT (s), median (IQR)	14–21	16.0 (14.2–17.1)	15.6 (13.8–16.6)	16.3 (15.0–17.5)	**<0.001**
Urea (mmol/L), median (IQR)	3.1–8.8	5.9 (4.3–8.5)	5.7 (3.8–7.4)	6.1 (4.4–9.2)	**0.016**
Creatinine (μmol/L), median (IQR)	41–81	70.0 (57.0–88.0)	65.0 (54.0–82.0)	74.0 (61.0–99.0)	**0.005**
AST (U/L), median (IQR)	13–35	32.0 (23.0–45.0)	29.0 (22.0–41.0)	34.5 (24.3–48.8)	**0.016**
ALT (U/L), median (IQR)	7–40	24.0 (16.0–39.0)	26.0 (18.0–40.0)	23.0 (15.3–36.5)	0.182
TBIL (μmol/L), median (IQR)	0–23	12.7 (9.7–16.4)	12.3 (9.4–15.4)	12.9 (10.0–17.5)	0.076
Albumin (g/L), mean (SD)	40–55	31.88 (4.62)	32.50 (4.21)	31.32 (4.89)	**0.026**
Sodium (mmol/L), median (IQR)	137–147	135.7 (131.9–138.5)	135.9 (133.0–139.4)	135.3 (130.3–138.0)	0.079
Kalium (mmol/L), median (IQR)	3.5–5.3	3.97 (3.65–4.38)	4.00 (3.66–4.41)	3.94 (3.64–4.38)	0.901
BNP (pg/mL), median (IQR)	0–100	99.0 (55.0–236.0)	78.0 (42.0–181.0)	116.0 (65.0–272.5)	**0.003**
Lactate dehydrogenase (U/L), median (IQR)	120–250	276.0 (229.8–375.0)	280.0 (223.0–364.8)	275.5 (230.0–387.0)	0.548
Creatine kinase (U/L), median (IQR)	40–200	84.0 (47.0–185.0)	71.0 (42.0–144.5)	103.0 (53.0–245.8)	**0.014**
Creatine kinase mb (ng/mL), median (IQR)	0–5	3.23 (2.39–4.45)	2.86 (2.12–3.95)	3.51 (2.62–5.33)	**<0.001**
Cardiac troponin I (ng/mL), median (IQR)	0.000–0.020	0.013 (0.006–0.030)	0.009 (0.005–0.018)	0.017 (0.009–0.048)	**<0.001**
IL6 (pg/mL), median (IQR)	0–10	39.14 (12.13–87.42)	25.74 (9.49–74.58)	47.54 (17.03–105.95)	**0.012**
Procalcitonin (ng/mL), median (IQR)	0–0.5	0.17 (0.08–0.86)	0.10 (0.07–0.40)	0.24 (0.09–1.59)	**0.004**
Treatments					
Antiviral therapy, *n* (%)		284 (94.0)	136 (95.8)	148 (92.5)	0.339
Antibiotic therapy, *n* (%)		247 (81.8)	106 (74.6)	141 (88.1)	**0.004**
Anticoagulation therapy, *n* (%)		205 (67.9)	97 (68.3)	108 (67.5)	0.979
Glucocorticoid therapy, *n* (%)		190 (62.9)	93 (65.5)	97 (60.6)	0.450
Immunomodulator therapy, *n* (%)		150 (49.7)	70 (49.3)	80 (50.0)	0.995
Respiratory support					0.079
Supplemental oxygen, *n* (%)		248 (82.1)	124 (87.3)	124 (77.5)	–
Noninvasive mechanical ventilation, *n* (%)		23 (7.6)	7 (4.9)	16 (10.0)	–
Invasive mechanical ventilation, *n* (%)		31 (10.3)	11 (7.8)	20 (12.5)	–
Length of hospital stays, median (IQR)		11.0 (8.0–15.0)	11.0 (8.3–14.0)	11.0 (8.0–16.0)	0.880
Outcomes					**<0.001**
Survival, *n* (%)		234 (77.5)	124 (87.3)	110 (68.7)	–
Death, *n* (%)		68 (22.5)	18 (12.7)	50 (31.3)	–

COVID-19, Coronavirus disease 2019; BMI, body mass index; ARDS, acute respiratory distress syndrome; AKI, acute kidney injury; SIRS, systemic inflammatory response syndrome; PH, potential of hydrogen; Pao2, partial pressure of oxygen; Paco2, partial pressure of carbon dioxide; PT, prothrombin time; APTT, activated partial thromboplastin time; INR, international normalized ratio; FDP, fibrin degradation products; FIB, fibrinogen; TT, thrombin time; AST, aspartate transaminase; ALT, alanine transaminase; TBIL, total bilirubin; BNP, B-type natriuretic peptide; IL6, interleukin-6. Bold values suggest *p* < 0.05.

### 3.2 Cox regression analysis

Based on univariable Cox regression analysis, risk factors associated with mortality in the young-old group included: respiratory rate, stomachache symptoms, symptoms of confusion, ARDS, AKI, respiratory weakness, lactate, FDP, urea, creatinine, AST, TBIL, lactate dehydrogenase, creatine kinase mb, IL6, procalcitonin, non-invasive mechanical ventilation and invasive mechanical ventilation (compared with supplemental oxygen) is related to death. Protective factors associated with mortality in the young-old group included: PaO_2_ and lymphocyte count.

The old-old group and the young-old group shared the following risk factors: respiratory rate, AKI, respiratory weakness, lactate, FDP, urea, creatinine, lactate dehydrogenase, creatine kinase mb, procalcitonin, non-invasive mechanical ventilation and invasive mechanical ventilation (compared with supplemental oxygen). The old-old group and the young-old group shared the following protective factors: PaO_2_ and lymphocyte count.

However, the following factors only showed significant impact on death in the old-old group: dyspnea (HR: 2.829, 95%CI: 1.571–5.093), PaO_2_/FiO_2_ (HR: 0.996, 95%CI: 0.994–0.999), PH (HR: 0.020, 95%CI: 0.001–0.627), heart rate (HR: 1.031, 95%CI: 1.014–1.049), INR (HR: 3.142, 95%CI: 1.139–8.669), platelet count (HR: 0.996, 95% CI: 0.992–1.000), cardiac troponin I (HR: 1.366, 95%CI: 1.161–1.606), acute cardiac injury (HR: 2.403, 95%CI: 1.369–4.219), white blood cell count (HR: 1.135, 95%CI: 1.070–1.203), neutrophil count (HR: 1.154, 95%CI: 1.092–1.220), albumin (HR: 0.909, 95%CI: 0.855–0.966), critical illness (HR: 72.980, 95%CI: 10.030–531.032) (compared with medium), chronic kidney disease (HR: 2.102, 95%CI: 1.116–3.959), diabetes (HR: 2.401, 95%CI: 1.311–4.397), glucocorticoid therapy (HR: 2.397, 95%CI: 1.198–4.798) (see Table 2).

**Table 2:** Cox regression analyses of risk factors for survival of young-old and old-old among senior COVID-19 patients.

Variables	Young-old COVID-19 patients (*N* = 142)		Old-old COVID-19 patients (*N* = 160)	
	HR (95% CI)	*P* value	HR (95% CI)	*P* value
Sex	1.424 (0.498–4.075)	0.510	1.248 (0.698–2.231)	0.455
Diabetes	0.918 (0.351–2.396)	0.860	2.401 (1.311–4.397)	**0.005**
Age	1.121 (0.916–1.370)	0.267	1.052 (1.000–1.107)	0.051
BMI	1.039 (0.883–1.222)	0.649	0.962 (0.887–1.043)	0.346
Temperature	0.820 (0.403–1.671)	0.585	0.800 (0.522–1.226)	0.306
Respiratory rate	1.192 (1.049–1.354)	**0.007**	1.162 (1.081–1.250)	**<0.001**
Mean arterial pressure	0.969 (0.926–1.014)	0.171	1.017 (0.999–1.035)	0.063
Heart rate	1.020 (0.987–1.054)	0.229	1.031 (1.014–1.049)	**<0.001**
Signs and symptoms				
Cough and expectoration	0.535 (0.169–1.692)	0.287	0.688 (0.309–1.534)	0.361
Fever	0.503 (0.113–2.242)	0.367	1.881 (0.676–5.234)	0.226
Feel cold	0.506 (0.066–3.857)	0.511	0.995 (0.422–2.347)	0.991
Chest pain	–	–	–	–
Chest tightness	0.627 (0.142–2.764)	0.538	0.537 (0.167–1.727)	0.297
Myalgia	0.230 (0.028–1.899)	0.172	–	–
Stomachache	6.931 (1.534–31.329)	**0.012**	–	–
Fatigue	0.392 (0.112–1.365)	0.141	0.700 (0.365–1.342)	0.283
Palpitate	–	–	–	–
Inappetence	0.514 (0.167–1.581)	0.245	0.984 (0.556–1.743)	0.956
Nausea and vomiting	0.406 (0.053–3.114)	0.386	0.869 (0.345–2.190)	0.766
Pharyngeal discomfort	–	–	0.466 (0.145–1.499)	0.200
Dyspnea	1.302 (0.476–3.561)	0.607	2.829 (1.571–5.093)	**0.001**
Stuffy and running nose	–	–	0.498 (0.069–3.612)	0.491
Confusion	10.466 (3.259–33.615)	**<0.001**	1.346 (0.670–2.704)	0.403
Complications				
Acute liver injury	1.165 (0.262–5.183)	0.841	0.800 (0.339–1.890)	0.611
ARDS	12.850 (1.641–100.613)	**0.015**	3.901 (0.940–16.181)	0.060
AKI	6.749 (2.411–18.890)	**<0.001**	2.853 (1.555–5.237)	**0.001**
SIRS	3.379 (0.749–15.236)	0.113	1.845 (0.921–3.697)	0.084
Acute cardiac injury	1.622 (0.562–4.680)	0.371	2.403 (1.369–4.219)	**0.002**
Respiratory weakness	17.484 (2.295–133.211)	**0.006**	9.454 (3.743–23.875)	**<0.001**
Comorbidities				
Chronic pulmonary disease	1.443 (0.467–4.462)	0.524	0.882 (0.375–2.073)	0.773
Chronic kidney disease	0.889 (0.116–6.809)	0.910	2.102 (1.116–3.959)	**0.021**
Hypertension	1.094 (0.412–2.902)	0.857	0.773 (0.444–1.345)	0.362
Chronic neurological disease	–	–	1.586 (0.789–3.190)	0.196
Cerebrovascular disease	2.194 (0.792–6.073)	0.131	1.078 (0.615–1.892)	0.792
Cardiovascular disease	2.593 (0.932–7.212)	0.068	1.647 (0.941–2.882)	0.081
Clinical classification				
Medium	Reference	Reference	Reference	Reference
Severe	–	–	7.253 (0.904–58.204)	0.062
Critical	–	–	72.980 (10.030–531.032)	**<0.001**
Laboratory findings				
PH	–	–	0.020 (0.001–0.627)	**0.026**
PaO2, mmHg	0.948 (0.911–0.988)	**0.010**	0.961 (0.939–0.983)	**0.001**
PaO2/FiO2, mmHg	0.996 (0.990–1.001)	0.083	0.996 (0.994–0.999)	**0.011**
PaCO2, mmHg	0.909 (0.827–0.999)	**0.048**	0.978 (0.931–1.028)	0.388
Lactate, mmol/L	2.092 (1.362–3.212)	**0.001**	2.199 (1.474–3.282)	**<0.001**
White blood cell count, ×10^9^/L	1.031 (0.897–1.186)	0.668	1.135 (1.070–1.203)	**<0.001**
Platelet count, ×10^9^/L	0.995 (0.988–1.001)	0.116	0.996 (0.992–1.000)	**0.046**
Hemoglobin, g/L	0.999 (0.975–1.023)	0.906	1.004 (0.990–1.018)	0.585
Lymphocyte count, ×10^9^/L	0.188 (0.051–0.701)	**0.013**	0.178 (0.071–0.451)	**<0.001**
Neutrophil count, ×10^9^/L	1.087 (0.955–1.237)	0.208	1.154 (1.092–1.220)	**<0.001**
D-dimer, ng/mL	–	–	–	–
PT, s	1.144 (0.848–1.542)	0.379	1.091 (0.988–1.205)	0.084
APTT, s	0.989 (0.926–1.057)	0.744	0.993 (0.958–1.030)	0.714
INR	3.066 (0.111–84.553)	0.508	3.142 (1.139–8.669)	**0.027**
FDP, μg/mL	1.034 (1.006–1.062)	**0.016**	1.010 (1.004–1.016)	**0.001**
FIB, g/L	1.031 (0.798–1.331)	0.818	1.061 (0.881–1.277)	0.531
TT, s	0.983 (0.873–1.106)	0.772	1.020 (0.997–1.043)	0.086
Urea, mmol/L	1.117 (1.071–1.164)	**<0.001**	1.105 (1.066–1.145)	**<0.001**
Creatinine, μmol/L	1.004 (1.002–1.006)	**<0.001**	1.006 (1.003–1.010)	**<0.001**
AST, U/L	1.016 (1.004–1.028)	**0.008**	1.003 (0.999–1.007)	0.110
ALT, U/L	1.003 (0.991–1.016)	0.618	1.003 (0.995–1.010)	0.468
TBIL, μmol/L	1.041 (1.015–1.069)	**0.002**	1.009 (0.984–1.035)	0.485
Albumin, g/L	0.931 (0.820–1.057)	0.269	0.909 (0.855–0.966)	**0.002**
Sodium, mmol/L	0.975 (0.911–1.042)	0.450	1.031 (0.991–1.073)	0.136
Kalium, mmol/L	1.627 (0.745–3.552)	0.222	1.387 (0.961–2.003)	0.081
BNP, pg./mL	1.000 (0.999–1.001)	0.926	–	–
Lactate dehydrogenase, U/L	1.006 (1.004–1.009)	**<0.001**	1.004 (1.003–1.006)	**<0.001**
Creatine kinase, U/L	–	–	–	–
Creatine kinase mb, ng/mL	1.036 (1.006–1.066)	**0.016**	1.055 (1.029–1.081)	**<0.001**
Cardiac troponin I, ng/mL	1.395 (0.510–3.817)	0.517	1.366 (1.161–1.606)	**<0.001**
IL6, pg./mL	1.002 (1.001–1.003)	**<0.001**	–	–
Procalcitonin, ng/mL	1.068 (1.023–1.115)	**0.003**	1.059 (1.030–1.088)	**<0.001**
Treatments				
Antiviral therapy	–	–	1.271 (0.395–4.092)	0.687
Antibiotic therapy	–	–	5.816 (0.802–42.186)	0.082
Anticoagulation therapy	2.814 (0.638–12.415)	0.172	1.868 (0.934–3.736)	0.077
Glucocorticoid therapy	1.598 (0.450–5.683)	0.469	2.397 (1.198–4.798)	**0.014**
Immunomodulator therapy	1.481 (0.538–4.078)	0.447	1.415 (0.796–2.514)	0.237
Respiratory support				
Supplemental oxygen	Reference	Reference	Reference	Reference
Noninvasive mechanical ventilation	6.963 (1.684–28.791)	**0.007**	3.462 (1.623–7.384)	**0.001**
Invasive mechanical ventilation	13.707 (4.465–42.079)	**<0.001**	8.410 (4.502–15.708)	**<0.001**

HR, hazard ratio; CI, confidence interval; “–”: event distributions are not suitable for COX regression analysis. Bold values suggest *p* < 0.05.

### 3.3 Kaplan Meier analysis

**Figure 1:**
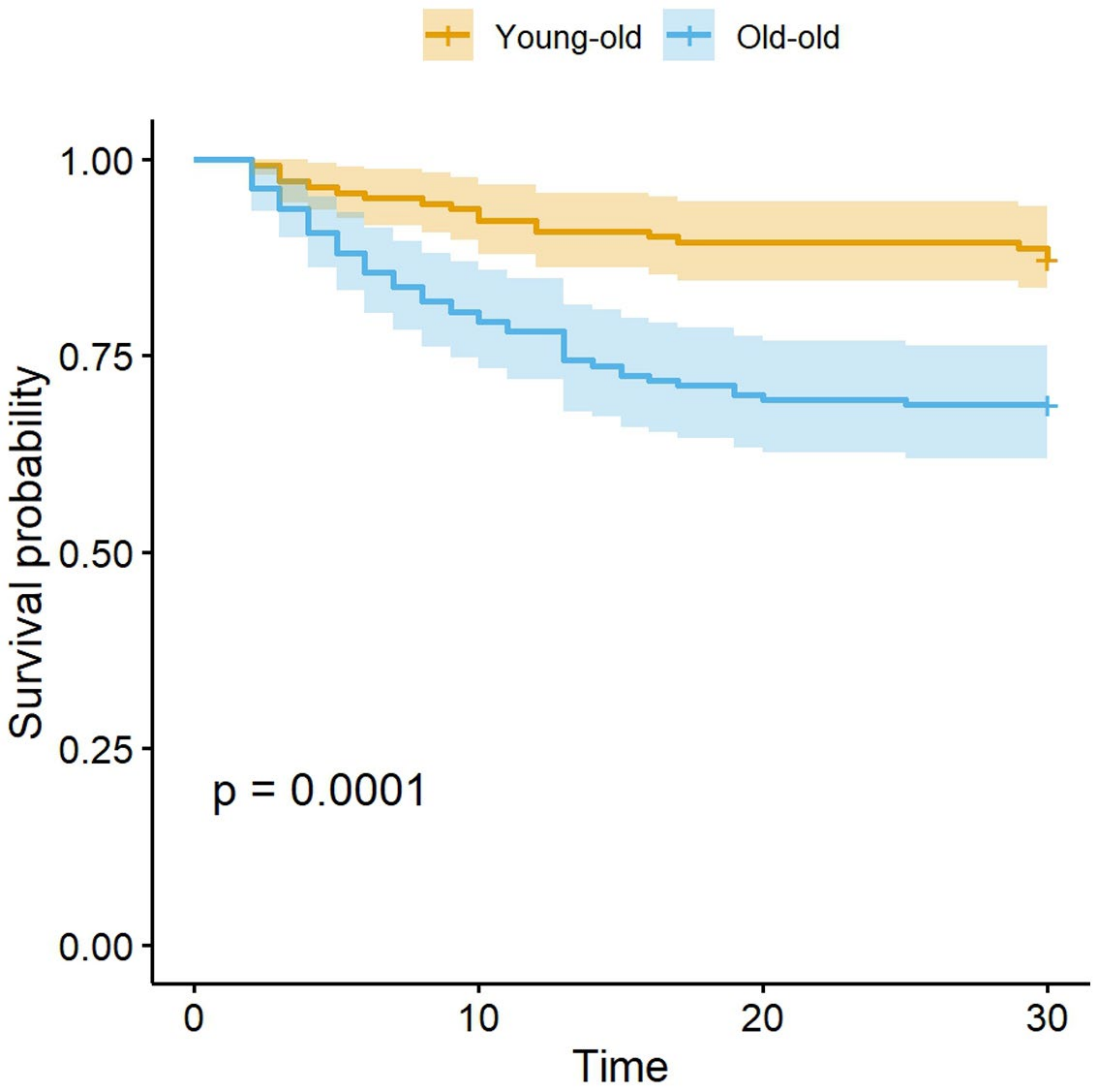
Kaplan–Meier survival curves displaying the survival probability in young-old and old-old COVID patients.

**Figure 2:**
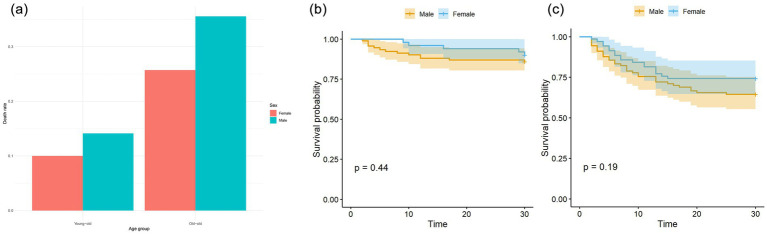
**(a)** Sex differences of mortality in young-old and old-old COVID patients. There was no significant sex difference in mortality in young-old group (*p* = 0.658), which was also seen in old-old group (*p* = 0.246). **(b)** Sex differences of Kaplan–Meier survival analysis in young-old COVID patients. **(c)** Sex differences of Kaplan–Meier survival analysis in old-old COVID patients.

The survival curve results showed that there was a significant difference in the survival curves between the young-old group and the old-old group (*p* = 0.0001) (see Figure 1). Subgroup analysis showed that no significant sex difference in mortality was found in either the young-old group or the old-old group (Figure 2a). There were no significant sex differences in the survival curves of each group (see Figures 2b,c).

## 4 Discussion

This study is the first to explore death-related risk factors and compare their similarities and differences among the young-old COVID-19 and old-old COVID-19 patients. Compared with young-old COVID-19 patients, the mortality of old-old patients is higher. There is a significant difference in the survival curves between the two groups. We also found some unique risk factors related to the death of old-old COVID-19 patients, including clinical symptoms, laboratory indicators, complications, comorbidities, which helps to improve the treatment of elderly COVID-19 patients to reduce mortality.

Compared with young-old COVID-19 patients, the mortality of old-old patients is higher, which is consistent with the study of Pijls (18). The expression of ACE2 receptor related to coronavirus infection is increased in old-old patients (19); elderly patients have lower immunity and are more likely to have aging-related diseases, such as diabetes, cardiovascular disease, chronic kidney disease, etc. (19). These factors may be related to the high mortality of old-old patients. In addition, for the first time, we found that there are significant differences in the survival curves of young-old COVID-19 patients and old-old patients. Previous studies only compared the difference in survival rates between the two populations (5, 8), while the survival curve can reflect both patient survival outcomes and survival time. It helps us better understand the differences in disease progression among elderly patients with COVID-19 at different age strata.

Consistent with previous studies, the univariate Cox proportional regression model of this study found that: physical signs (respiratory rate) (20), laboratory parameters (PaO_2_, lactate, lymphocyte count, FDP, urea, creatinine, LDH, Creatine kinase mb, procalcitonin) (20–24), complications (respiratory weakness, acute kidney injury) (20, 23), treatment (non-invasive mechanical ventilation, invasive mechanical ventilation) (25) had an impact on the survival of both young-old and old-old patients. It is worth noting that compared with young-old COVID-19 patients, dyspnea symptoms, PH, PaO_2_/FiO_2_, heart rate, INR, cardiac troponin I, acute cardiac injury, white blood cell count, neutrophil count, platelet count, albumin, critical illness, chronic kidney disease, combined diabetes, and glucocorticoid therapy factors were only associated with death in old-old patients.

Dyspnea, decreased PaO_2_/FiO_2_, and decreased PH may be manifestations of impaired respiratory function (26). The COVID-19 virus mainly affects the lungs, and the old-old patients often suffer from chronic obstructive pulmonary disease. The impaired oxygenation caused by the COVID-19 virus will bring greater challenges to the lung function of the old-old patients. An increased heart rate indicates an increased risk of ARDS related to COVID-19, which is associated with an increased mortality rate (26). INR and platelet count reflects coagulation function and is often abnormal in old-old patients (27, 28). Excessive INR values and decreased platelet count are associated with an increased mortality in COVID-19 patients (29, 30). An increase in cardiac troponin I indicates myocardial damage. Old-old patients are more likely to suffer from acute heart damage after being infected with the new coronavirus (31). Increased white blood cell counts and neutrophil counts may indicate that the patient has a bacterial infection (31). Old-old patients have low immunity, which may be related to a higher mortality rate. Albumin is an indicator of nutritional status. Older patients are more likely to be malnourished and have a poorer ability to resist infection, which can lead to a poor prognosis (32). Critically ill COVID-19 patients are more likely to die, and the condition of old-old patients is more serious, and the mortality is higher (8, 31). The prevalence of chronic kidney disease is higher in the old-old than in the younger ones, and renal function gradually deteriorates with age (33). Old-old patients with chronic kidney disease have more severe conditions and worse prognosis after being infected with SARS-COV2 (34). Previous studies have found that diabetes is a risk factor for death in elderly COVID-19 patients (35). The prevalence of diabetes is higher in old-old patients, and long-term hyperglycemia is more harmful to the body. The use of glucocorticoids in older patients, especially those with severe illness, must be approached with caution, as their administration may correlate with increased mortality rates. Future studies should aim to adjust for illness severity when evaluating treatment outcomes.

Subgroup analysis showed that there was no sex difference in mortality in both young-old and old-old patients, and there was still no difference in the survival curves between men and women. Previous studies have found that male sex is a risk factor for death in elderly COVID-19 patients (36), but these studies only explored the effect of gender on death in the overall elderly COVID-19 patients and did not rule out the confounding effect of age on gender.

Our findings directly inform COVID-19 management for elderly patients in three key areas: (1) risk stratification: the 75-year threshold identifies patients requiring heightened monitoring (vital signs, daily cardiac markers); dyspnea in old-old patients should trigger immediate oxygenation assessment and cardiology consultation. (2) Treatment modifications: glucocorticoid use in patients ≥75 years requires careful risk–benefit evaluation; prophylactic anticoagulation should be prioritized given elevated D-dimer levels. (3) Resource allocation: old-old patients merit higher nurse-to-patient ratios; hospitals should pre-allocate ventilators for this high-risk population during surges.

This study has some limitations. First, this study used a convenient sampling method, and the samples came from a tertiary hospital in one city, which lacks the representativeness of the national situation. Second, the sample size is small, and the follow-up time of the study is short. The long-term outcomes of patients after discharge can be continuously monitored, and a large sample of multiple centers across the country can be used to verify the findings of this study in the future. Moreover, important confounders were not captured, including: socioeconomic status and healthcare access, detailed medication histories (e.g., immunosuppressants), community-level transmission rates, genetic factors influencing COVID-19 susceptibility. While 75 years is clinically meaningful in our setting, we acknowledge variations may exist in populations with different life expectancies. We recommend context-specific validation when applying these findings to regions with younger/older population pyramids. Vaccination status was not available for analysis due to inconsistent documentation during the study period. This is a significant limitation given the established protective effects of COVID-19 vaccination. However, several contextual factors should be noted: (1) China’s vaccination campaign achieved >90% primary series coverage by late 2022, potentially reducing inter-individual variability; (2) our hospital primarily admitted severe cases where vaccine effectiveness may be attenuated; and (3) the Omicron variant dominated during our study period, when vaccine protection against severe outcomes was maintained but with reduced effectiveness against infection. Future studies should prioritize systematic vaccination data collection. Moreover, our sample size limited the number of variables we could adjust for in multivariable analyses (approximately 1 variable per 10 events). This constrained our ability to fully adjust for all potential confounders simultaneously, larger sample size is warranted for future studies.

## 5 Conclusion

For the first time, this study found that acute cardiac injury, diabetes, glucocorticoid therapy, dyspnea symptoms, INR and other factors are unique factors related to mortality risk in old-old COVID-19 patients. Older COVID-19 patients have a higher mortality and a significant different survival curve than young-old COVID-19 patients. These findings will help guide the precise treatment of elderly COVID-19 patients at different ages and improve the survival rate of senior patients. Our findings reflect outcomes during China’s Omicron-dominant period (December 2022–March 2023) with high population immunity from prior exposure. Caution is warranted when applying results to: populations with different variant distributions; regions with lower vaccination rates; healthcare systems with differing resource availability.

## Data Availability

The raw data supporting the conclusions of this article will be made available by the authors, without undue reservation.
